# *Zingiber cassumunar* Roxb. Essential Oil-Loaded Electrospun Poly(lactic acid)/Poly(ethylene oxide) Fiber Blend Membrane for Antibacterial Wound Dressing Application

**DOI:** 10.3390/membranes11090648

**Published:** 2021-08-24

**Authors:** Pattawika Sinsup, Veerawat Teeranachaideekul, Arthit Makarasen, Laemthong Chuenchom, Pongthep Prajongtat, Supanna Techasakul, Peerada Yingyuad, Decha Dechtrirat

**Affiliations:** 1 Department of Materials Science, Faculty of Science, Kasetsart University, Bangkok 10900, Thailand; pattawika.s@ku.th (P.S.); fscipop@ku.ac.th (P.P.); 2 Department of Pharmacy, Faculty of Pharmacy, Mahidol University, Bangkok 10400, Thailand; veerawat.tee@mahidol.edu; 3 Laboratory of Organic Synthesis, Chulabhorn Research Institute, Bangkok 10210, Thailand; arthit@cri.or.th (A.M.); supanna@cri.or.th (S.T.); 4 Division of Physical Science, Center of Excellence for Innovation in Chemistry, Faculty of Science, Prince of Songkla University, Songkhla 90110, Thailand; laemthong.c@psu.ac.th; 5 Department of Chemistry, Faculty of Science, Kasetsart University, Bangkok 10900, Thailand; 6 Specialized Center of Rubber and Polymer Materials for Agriculture and Industry (RPM), Faculty of Science, Kasetsart University, Bangkok 10900, Thailand

**Keywords:** controlled release, electrospinning, essential oil, fibrous membrane, wound dressing, *Zingiber cassumunar* Roxb.

## Abstract

The essential oil from *Zingiber cassumunar* Roxb. (*Plai*) has long been used in Thai herbal remedies to treat inflammation, pains, sprains, and wounds. It was therefore loaded into an electrospun fibrous membrane for use as an analgesic and antibacterial dressing for wound care. The polymer blend between poly(lactic acid) and poly(ethylene oxide) was selected as the material of choice because its wettability can be easily tuned by changing the blend ratio. Increasing the hydrophilicity and water uptake ability of the material while retaining its structural integrity and porosity provides moisture balance and removes excess exudates, thereby promoting wound healing. The effect of the blend ratio on the fiber morphology and wettability was investigated using scanning electron microscopy (SEM) and contact angle measurement, respectively. The structural determination of the prepared membranes was conducted using Fourier-transform infrared spectroscopy (FTIR). The release behavior of (E)-1-(3,4-dimethoxyphenyl) butadiene (DMPBD), a marker molecule with potent anti-inflammatory activity from the fiber blend, showed a controlled release characteristic. The essential oil-loaded electrospun membrane also showed antibacterial activity against *S. aureus* and *E. coli*. It also exhibited no toxicity to both human fibroblast and keratinocyte cells, suggesting that the prepared material is suitable for wound dressing application.

## 1. Introduction

*Zingiber cassumunar* Roxb., named *Plai* in Thailand, is a medicinal plant that has long been used in Thai traditional herbal remedies for pain, sprains, inflammation, wounds, skin diseases, asthma, and rheumatism [1,2,3]. The essential oil and extract from the fresh rhizome of *Zingiber cassumunar* Roxb. have been reported to exhibit local anesthetic and analgesic effects [4,5], reduce pain and inflammation [6,7,8,9], and inhibit the growth of fungi and bacteria [10,11,12]. Due in part to its reported medical uses and pharmacological activities, *Zingiber cassumunar* Roxb. is a good candidate for development as a medical or health care product.

Wound dressing plays an essential role in accelerating the wound healing process. The ideal dressing should prevent bacterial infection, remove excess exudates, allow the ease of gas exchange, and maintain moisture balance on the wound bed [13,14]. Compared with typical dressings such as gauzes, lint, bandages, and cotton wool, nanofibers are more attractive, owing to their large specific surface area, high porosity, and excellent pore interconnectivity. These extraordinary characteristics exert several advantages for wound healing, such as assisting cell attachment and proliferation as well as facilitating the permeability of moisture and gas, which are beneficial for cell growth and allowing the absorption of additional exudates containing nutrients for bacterial growth. In addition, nanofiber scaffolds closely mimic the structure of the extracellular matrix (ECM) [15,16,17]. Therefore, nanofibers are promising candidates for wound dressing materials as they provide a suitable environment for wound healing.

In recent years, both natural and synthetic polymers have been used to prepare nanofiber-based wound dressings via the electrospinning process. Among these different polymers, poly(lactic acid) (PLA), an FDA-approved synthetic biodegradable polymer derived from renewable resources, is of particular interest. Due to its biodegradability, biocompatibility, good mechanical properties, and low cost, PLA has become a candidate of choice in bio-related applications [18,19,20]. The hydrophobic nature of PLA could render better interaction with lipophilic drugs or plant essential oils. However, this property could also reduce cellular interactions and limit exudate uptake capability. Therefore, PLA needs to be modified in order to adjust its wettability property while retaining its structural integrity and porous morphology. In bulk modification, hydrophilic polymers such as polyethylene oxide (PEO) are usually incorporated into the electrospinning solution [21,22,23,24]. PEO is biodegradable, biocompatible, non-toxic, and FDA-approved. Thus, it is considered a suitable choice to produce PLA-based composite nanofibers. It has been reported that PLA mixed with PEO could successfully be electrospun, resulting in nanofibers with a smooth surface, high porosity, and enhanced hydrophilicity while maintaining structural integrity when compared with pure PLA nanofibers [21]. In a previous study, the incorporation of rapamycin, a water-insoluble antibiotic and antiproliferative agent, into the PLA and PEO blend solution in the appropriate ratios produced smooth and uniform nanofibers with high encapsulation efficiency [25]. The blended PLA/PEO nanofiber was also shown to manipulate and control the release of loaded natural and synthetic compounds [25,26]. In addition, the PLA/PEO fiber blend loaded with grape seed extract (GSE) enhanced fibroblast cell adhesion and proliferation in comparison with the PLA/GSE nanofiber as a result of increased hydrophilicity [27].

**Scheme 1:**
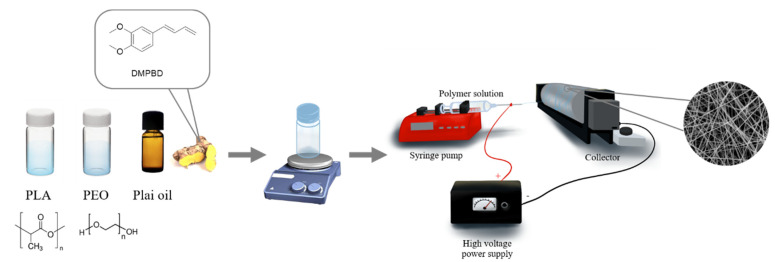
Schematic representation for the fabrication of the *Plai* essential oil-loaded electrospun PLA/PEO fibrous membrane.

The electrospun nanofiber loaded with *Plai* oil has been studied previously by Tonglairoum et al. using polyvinylpyrrolidone (PVP), a hydrophilic polymer, blended with 2-hydroxypropyl-β-cyclodextrin (HPβCD) [28]. It was found that a maximum of 20% of *Plai* oil could be loaded into the nanofiber with high entrapment efficiency. However, entrapment efficiency decreased markedly when the incorporated amount of *Plai* oil was increased to 30%. These findings were primarily due to less hydrophilic-hydrophobic interaction between the PVP polymer and *Plai* oil. In addition, the PVP nanofiber showed a relatively short shelf life of up to one week before they fused together, which could be a result of the hygroscopic nature of PVP. PLA has been proven to be a promising matrix to improve the entrapment efficiency of *Plai* oil as well as prolong nanofiber shelf life [29]. Therefore, this work incorporated *Plai* essential oil into a PLA/PEO fiber blend. PEO was blended with PLA to produce a matrix with enhanced hydrophilicity, resulting in the increased water uptake capacity of the fiber. The *Plai* essential oil-loaded fiber blend was prepared through electrospinning (Scheme 1). Characterization of the prepared materials was carried out using various techniques. In vitro release, antibacterial, and cytotoxicity tests were also performed to demonstrate the potential of using the prepared materials as wound dressings.

## 2. Materials and Methods

### 2.1. Materials

*Plai* essential oil was obtained from Thai China Flavours & Fragrances Industry Co., Ltd. (Bangkok, Thailand). PEO (Mw ~ 100,000) and PLA (Mw ~ 60,000) were acquired from Sigma Aldrich. Dulbecco’s modified Eagle’s medium (DMEM, Gibco^®^, MD, USA) with 10% fetal bovine serum (FBS, Gibco^®^), 1 mM sodium pyruvate (Gibco^®^), and penicillin/streptomycin (Gibco^®^) were supplied by Life Technologies. In addition, (3-(4,5-dimethylthiazol-2-yl)-5-(3-carboxymethoxyphenyl)-2-(4-sulfophenyl)-2H-tetrazolium inner salt was from obtained Promega Corporation. All buffer salts and organic solvents were of analytical grade from Merck.

### 2.2. Analysis of Plai Essential Oil

The chemical constituents and DMPBD content in *Plai* essential oil were determined using GC/MS (QP2020, Shimadzu, Japan). Briefly, the analysis was performed using a capillary column (SH-Rxi-5Sil MS; 30 m length; 0.25 mm ID; 0.25 μm film thickness, Shimadzu, Japan) with an injection volume of 1 µL. The temperature of the injection port was fixed at 200 °C. The temperature of the oven was increased from 60 °C to 150 °C within 18 min, from 150 °C to 180 °C within 3 min, and then kept at 180 °C for 5 min. The mass scan was operated using the electron impact ionization mode over the mass range of 45–400 amu. DMPBD was isolated and characterized as previously described by Wongkanya et al. [29]. Briefly, *Plai* oil was loaded onto the silica gel column and then eluted with hexane/ethyl acetate (95:5 *v*/*v*). DMPBD, as a colorless oil, was obtained after solvent evaporation under reduced pressure.

### 2.3. Fabrication of Electrospun Fiber Blend Membranes

PLA/PEO solutions were prepared in DCM/DMSO (8:2 *v*/*v*) at 8%, 10%, and 12% *w*/*v*. The weight ratios of PLA and PEO were 9:1, 8:2, and 7:3 *w*/*w*. The effects of polymer concentration and PLA/PEO weight ratio on fiber morphology were studied. *Plai* oil at 30% (*w*/*w*, to the polymer content) was added into the polymer solutions and then thoroughly mixed. The prepared solutions were filled in a 10 mL glass syringe connected with a blunt needle (20-gauge). The syringe was assembled to the infusion pump, and the polymer solution was delivered at 0.5 mL/h. The electrospinning was operated using a high-voltage power supply (ES30P-5W, GAMMA, Ormond Beach, FL, USA) at 20 kV with the collector/needle tip distance of 15 cm. The experiments were carried out at 25 °C and 40% RH. The electrospun fibers were collected on a spinning drum and thereafter stored in a desiccator in the dark until further study.

### 2.4. Materials Characterization

#### 2.4.1. Scanning Electron Microscopy

The electrospun membranes were mounted to the stubs. The samples were subsequently coated with gold using a sputter coater. Then, the fiber morphology was visualized using a scanning electron microscope (Quanta 450, FEI, Eindhoven, The Netherlands) at an accelerating voltage of 15 kV. Fiber diameters from the SEM micrographs were analyzed using the ImageJ software (NIH), and the distribution and average of fiber diameters were determined from 100 random fibers.

#### 2.4.2. Contact Angle Measurement

Water contact angle measurement was conducted by an optical contact angle measuring system (Dataphysics OCA 20). A 5 µL water droplet was dispensed onto the fiber mat, and the corresponding image of the water droplet was taken. In total, 10 different positions on the fiber mat were tested, and the average contact angle was calculated.

#### 2.4.3. ATR-FTIR

The pristine fiber, fiber blend, and *Plai* oil-loaded fiber blend membranes were analyzed by a Fourier-transform infrared spectrometer (Perkin-Elmer Spectrum One FTIR, USA). The spectra were collected from 4000 cm^−1^ to 650 cm^−1^ with 64 scans and a resolution of 4 cm^−1^.

#### 2.4.4. Entrapment Efficiency of DMPBD in the Fiber Blend

Solvent extraction of DMPBD from the fiber blend was performed by placing the fiber membrane in a sealed glass vial containing 20 mL hexane. After continuously agitating for 6 h, the extraction solvent was collected and the amount of DMPBD extracted was analyzed by GC/MS. The entrapment efficiency was calculated using Equation (1):

EE% = (w_t_/w_i_) × 100 (1)

where w_t_ is the weight of DMPBD extracted from the fiber blend and w_i_ is the initial weight of DMPBD loaded into the fiber blend.

### 2.5. In Vitro Release

The in vitro release of DMPBD from the fiber membrane was conducted in PBS solution (pH 7.4) at ambient temperature. The pre-weighed fiber samples with a diameter of 16 mm were placed onto a regenerated cellulose membrane and mounted between the donor and receptor chambers of Franz diffusion cell. At specific time intervals of 0.17, 0.33, 0.5, 0.67, 0.83, 1, 2, 4, 6, 8, 10, 12, 24, and 48 h, 1 mL of solution was withdrawn and directly replaced with an equal amount of fresh PBS solution. The collected samples were subsequently extracted with 500 μL hexane twice. After solvent extraction, the hexane layer was collected, combined, and evaporated. The obtained residue was redissolved in hexane, and the amount of DMPBD released was quantified using GC/MS.

Release kinetics were assessed using the Ritger–Peppas equation [30], which is often used to describe the release from the polymeric system. An initial 60% of the cumulative release data were fitted to Equation (2):

M_t_/M_∞_ = kt^*n*^ (2)

where M_t_/M_∞_ is the fraction of compound released at time t, k is the rate constant, and n is the release exponent, which identifies the release mechanism. When *n* ≤ 0.5, the release is governed by a Fickian diffusion mechanism. When *n* ≥ 1.0, the release mechanism follows the case II transport. The mechanism lies between the previous means for 0. 5 < *n* < 1 and is considered an anomalous non-Fickian transport.

### 2.6. In Vitro Antibacterial Test

The in vitro antibacterial activity of the fiber blend was tested using a disk diffusion method. *Staphylococcus aureus* (*S. aureus*) (ATCC 25923) and *Escherichia coli* (*E. coli*) (ATCC 25922) were chosen as representatives for gram-positive and gram-negative bacterial strains frequently involved in wound infections [31]. These bacteria were cultured at 37 °C for 24 h. Subsequently, the bacterial suspension of 1 × 10^8^ CFU/mL was spread over the Mueller–Hinton agar (MHA) plate. The fiber membranes (Ø 6 mm) were sterilized with UV light for 30 min prior to testing. Then, the fiber membranes were mounted on the agar plates and incubated at 37 °C for 24 h. The antibacterial activity of the fiber blend membranes was thereafter calculated from the diameter of the clear inhibition zone. The experiments were conducted in triplicate, and the results were reported as the mean ± standard deviation.

### 2.7. In Vitro Cytotoxicity Test

Human primary fibroblast cells (Normal, Human, Adult (HDFa), ATCC^®^ PCS-201-012™, Manassas, VA, USA) and immortalized human keratinocytes (HaCaT, human keratinocyte cells, ATCC^®^ Number PCS-200-011™, 300493, CLS, Eppelheim, Germany) were cultured in Dulbecco’s modified Eagle’s medium (DMEM) with 10% fetal bovine serum (FBS), 1 mM sodium pyruvate, 100 U/mL penicillin, and 100 µg/mL streptomycin. The cells were cultured at 37 °C and 5% CO_2_ and were serially passaged at 70–80% confluence. Then, the experiments were performed with subconfluent cells at passage three in the proliferation phase.

In this test, cytotoxicity was evaluated by MTS assay. The cultured cells were seeded 1 day prior to the test in 96-well plates (10,000 cells/well for fibroblast and 8000 cells/well for keratinocyte). The fiber membranes were immersed in PBS for 24 h to obtain the extraction media at 0.63, 1.25, 2.5, and 5 mg/mL. The extract was then filtered through the 0.22 µm syringe filter. Then, cells were exposed to the extraction media at the different concentrations. PBS was used as a negative control for the test. The supernatant was removed after 24 h of incubation, and cells were rinsed twice with PBS. Fresh media (100 µL) containing MTS (20 µL) was added in each well plate. Then, cells were incubated at 5% CO_2_ at 37 °C for another 3 h. For viable cells, the colorless MTS reagent was converted into a soluble-colored 1-(4,5-dimethylthiazol-2-yl)-3,5-diphenylformazan product, which absorbed light at 490 nm. Cell viability was then calculated based on a change in absorbance at 490 nm compared to the negative control.

### 2.8. Statistical Analysis

The experimental data are reported as mean ± standard derivation (SD). The results were analyzed by one-way ANOVA. The *p*-values of less than 0.05 were statistically accepted as significant.

## 3. Results and Discussion

### 3.1. Fabrication and Characterization of Fiber Blend Membranes

**Figure 1:**
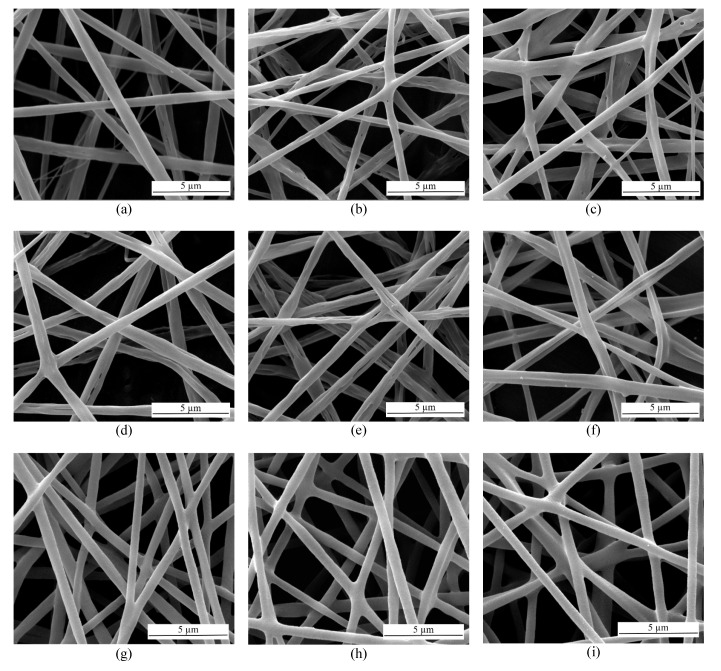
SEM images of the PLA/PEO fiber blend prepared at different polymer concentrations and PLA/PEO ratios: (**a**) 8% PLA/PEO (90:10), (**b**) 8% PLA/PEO (80:20), (**c**) 8% PLA/PEO (70:30), (**d**) 10% PLA/PEO (90:10), (**e**) 10% PLA/PEO (80:20), (**f**) 10% PLA/PEO (70:30), (**g**) 12% PLA/PEO (90:10), (**h**) 12% PLA/PEO (80:20), and (**i**) 12% PLA/PEO (70:30).

**Figure 2:**
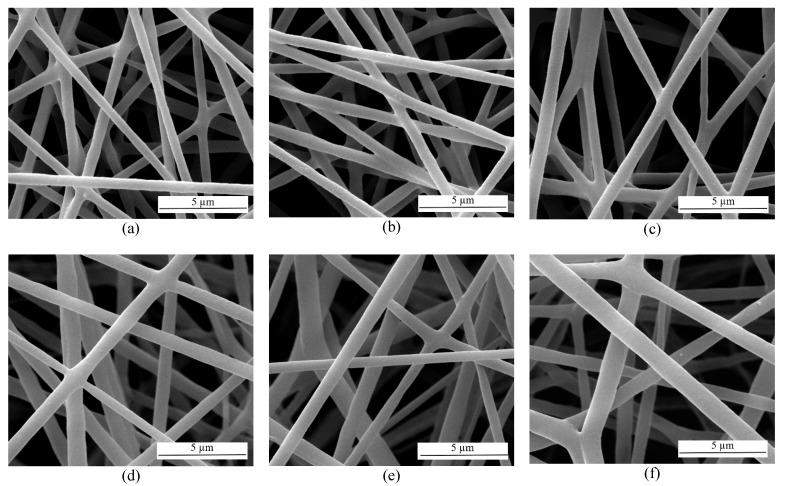
SEM images of the *Plai* oil-loaded fiber blend prepared at 12% polymer concentration with different PLA/PEO ratios: (**a**) PLA/PEO (90:10) + *Plai* oil, (**b**) PLA/PEO (80:20) + *Plai* oil, (**c**) PLA/PEO (70:30) + *Plai* oil, and their corresponding images after three months storage: (**d**) PLA/PEO (90:10) + *Plai* oil, (**e**) PLA/PEO (80:20) + *Plai* oil, and (**f**) PLA/PEO (70:30) + *Plai* oil.

The oil-free PLA/PEO fiber blend membranes were successfully prepared through the electrospinning of the PLA/PEO mixture solutions at the polymer concentrations of 8%, 10%, and 12% *w*/*v*. The weight ratios of PLA and PEO were 9:1, 8:2, and 7:3 *w*/*w*. The effects of polymer concentration and PLA/PEO weight ratio on fiber size and morphology were studied by SEM. As shown in Figure 1, the fibers prepared at the polymer concentrations of 8% and 10% exhibited a grooved and wrinkled structure at every blend ratio. The formation of this secondary surface morphology is attributed to the fast solvent evaporation (dichloromethane) at the early stage of electrospinning, followed by phase separation and the creation of tiny holes on the fiber surfaces. After the elongation and solidification of the polymer jet, the created voids turned themselves into grooves and wrinkles on the fiber surfaces [32]. On the contrary, at 12% of the polymer blend, uniform fibers with a smooth surface were obtained. This suggests that the low polymer concentration is preferable for the formation of grooves and wrinkles, as previously reported by Liu et al. [33]. Hence, 12% of the polymer blend concentration was chosen to prepare the essential oil-loaded fibrous membranes for further investigation. There are no obvious differences in the SEM images at the same polymer concentration, suggesting that the PLA/PEO weight ratio had no apparent effect on fiber morphology. The *Plai* oil-loaded fiber blend membranes at different PLA/PEO ratios were obtained by electrospinning the polymer blend solutions (12% *w*/*v*) incorporated with *Plai* oil. As seen in Figure 2a–c, the oil-loaded fibers had a round shape with smooth surfaces similar to those without *Plai* oil, revealing that the loading of *Plai* oil did not affect fiber morphology.

The average fiber diameters were determined from the SEM micrographs and are summarized in Table 1. At the same polymer concentration but different PLA/PEO blend ratios, the fiber diameters were not statistically different, suggesting that the blend ratio exerted no apparent effect on the fiber size. When the polymer concentration increased, the mean diameter increased as a result of the increase in molecular entanglement and viscosity of the solution. On the contrary, the mean diameters of the *Plai* oil-loaded fiber blend membranes were relatively smaller than those without *Plai* oil. This is possibly due to the decrease in solution viscosity after incorporating *Plai* oil into the blend solution.

**Figure 3:**
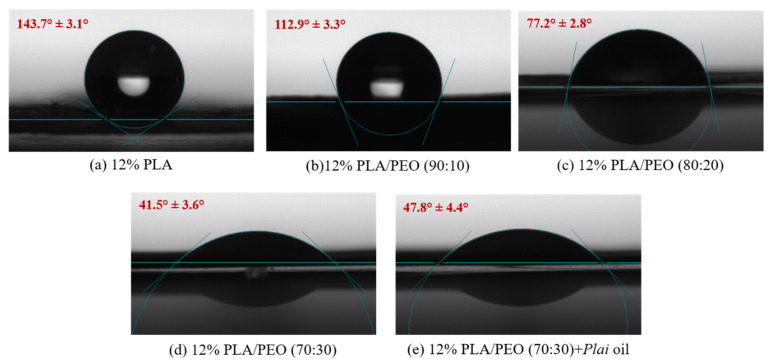
Water contact angles of (**a**) 12% PLA, (**b**) 12% PLA/PEO (90:10), (**c**) 12% PLA/PEO (80:20), (**d**) 12% PLA/PEO (70:30), and (**e**) 12% PLA/PEO (70:30) + *Plai* oil fiber membranes.

The water contact angle measurement was performed to investigate the surface wettability of the prepared fibrous membranes after blending PLA with PEO at different weight ratios. As evidenced by the water contact angle measurement in Figure 3, the average contact angle of the fibers tended to decrease with the increasing amount of PEO. The mean contact angles of the PLA fiber and the blend at the PLA/PEO weight ratios of 90:10, 80:20, and 70:30 were 143.7° ± 3.1°, 112.9° ± 3.3°, 77.2° ± 2.8°, and 41.5° ± 3.6°, respectively. As the weight amount of PEO increased, the contact angle decreased, similar to the previous report by Athanasoulia et al. [34]. The contact angle of the blend at the PLA/PEO ratio of 70:30 was the lowest, indicating the most hydrophilic surface and suggesting the highest water uptake ability. Increasing those properties is particularly important for the dressing application as they enhance the wound exudate absorption, thereby promoting the wound healing process. Hence, the blend at the 70:30 weight ratio was chosen for further study. After loading *Plai* oil into the fiber blend, the contact angle increased to 47.8° ± 4.4°, indicating a slightly more hydrophobic surface due to the lipophilic nature of *Plai* oil. However, the fiber blend membrane still exhibited high surface wettability, even in the presence of *Plai* oil.

Although the hydrophilic *Plai* oil-loaded PVP electrospun fiber mat has already been established [28], it has a limited storage life of less than one week due to the hygroscopic property of PVP, which causes the fibers to melt and fuse together. On the contrary, the *Plai* oil-loaded fiber blend membrane prepared within this work was found to be stable for at least three months. No change in fiber morphology was found in the SEM images of the membranes after three months of storage (Figure 2d–f). This indicates that the PLA/PEO blend is an excellent polymeric matrix for fabricating hydrophilic fiber-based wound dressing. Its physical stability against moisture in the air remained high, even after long-term storage. Furthermore, its wettability can also be promptly tailored by altering the blend ratio.

To further confirm the suitability of the PLA/PEO blend as a platform for *Plai* oil loading, the entrapment efficiency of *Plai* oil was evaluated. The entrapment efficiency was determined based on the amount of DMPBD entrapped within the fiber blend. The DMPBD underwent solvent extraction from the *Plai* oil-loaded fiber blend membrane and was then quantified by GC/MS. The entrapment efficiency was found to be as high as 94.6 ± 3.2%, indicating that the electrospinning did not cause any apparent changes to the bioactive compound. This high entrapment efficiency can be primarily attributed to the excellent miscibility of *Plai* oil with PLA [29]. In the previous report on the *Plai* oil-loaded HPβCD/PVP nanofiber, the entrapment efficiency was found to be only 55.5% [28]. This further confirms that the PLA/PEO blend is a good matrix for *Plai* oil loading.

**Figure 4:**
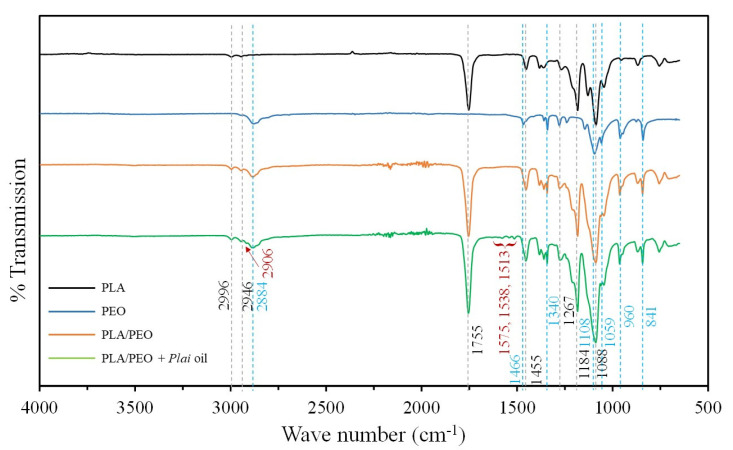
FTIR spectrum of pristine PLA, pristine PEO, the PLA/PEO fiber blend, and the oil-loaded PLA/PEO fiber blend membranes.

Structural determination and compatibility between *Plai* oil and the blend were studied using Fourier-transform infrared spectroscopy (FTIR). As shown in Figure 4, the PLA fibrous membrane exhibited strong characteristic peaks of C=O, stretching of the carbonyl group at 1755 cm^−1^, and corresponding bending at 1267 cm^−1^. It also showed strong peaks of C–O–C as well as stretching of the ester entity at 1184 cm^−1^ and 1088 cm^−1^. C–H stretching peaks at 2996 cm^−1^ and 2946 cm^−1^ as well as C–H bending at 1455 cm^−1^ are also typically found in the PLA spectrum. The pristine PEO showed typical C–H stretching peaks of the methylene group at 2948 cm^−1^ and 2884 cm^−1^, C–H bending at 1466 cm^−1^, CH_2_ wagging at 1340 cm^−1^, and CH_2_ rocking and twisting at 960 cm^−1^. Its spectrum also exhibited the C–O–C stretching peaks of the ether moiety at 1108 cm^−1^ and 1059 cm^−1^, and bending at 841 cm^−1^. As a blend, the PLA/PEO fibrous membrane showed all of the characteristic peaks of the pristine PLA and PEO at the same positions, suggesting that blending did not change the chemical structures of both polymers. After incorporating *Plai* oil into the fiber blend membrane, all of the characteristic peaks of PLA and PEO were still present in the same position as the blend without oil loading, suggesting that *Plai* oil also did not affect the chemical structures of both polymers. Furthermore, it also means that the interactions between *Plai* oil and the polymer blend were not strong enough to shift the peak positions. As the majority of chemical components in *Plai* oil are monoterpenes, their typical C–H stretching and bending peaks overlapped with those of PLA and PEO. This resulted in a slight increase in peak intensity at those stretching and bending regions after incorporation of *Plai* oil. The additional C–H stretching peak can also be observed at around 2906 cm^−1^ after the oil loading. Furthermore, the oil-loaded fiber blend membrane also showed vibrational peaks of aromatic constituents between 1575 cm^−1^ and 1513 cm^−1^ [29,35]. This confirms the presence of DMPBD in the fiber blend membrane.

### 3.2. In Vitro Release Study

**Figure 5:**
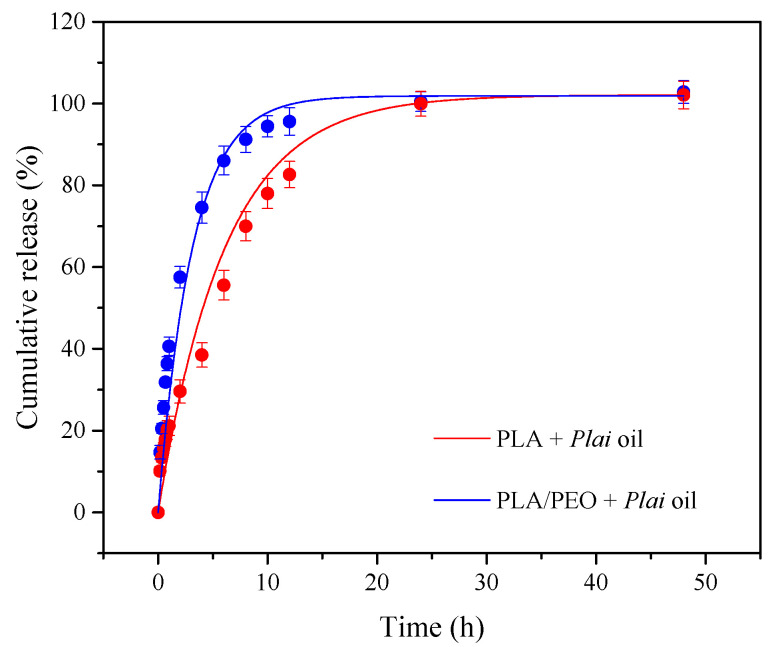
Cumulative release of DMPBD from the *Plai* oil-loaded fibrous membranes.

The cumulative release of DMPBD from the essential oil-loaded PLA and PLA/PEO (70:30) fiber blend membranes is depicted in Figure 5. The release of DMPBD for both membranes was fast at the initial state, mainly due to the burst release of the loosely bound DMPBD located at or near the essentially large fiber surfaces. After the initial fast desorption, a sustained release of the DMPBD embedded within the fibers was observed. The release at this region gradually decreased until reaching equilibrium.

At the early stage of release, the fiber blend showed a steeper slope than that of the PLA-based membrane. Afterward, the release of the marker compound from the blend increased continuously and nearly reached a plateau at an interval of 12 h. On the contrary, only ca. 80% of DMPBD was discharged from the PLA fibrous membrane at 12 h and almost 100% was released after 24 h. These findings reveal that the fiber blend exhibited a faster discharge of DMPBD. The higher hydrophilicity and water uptake ability of the blend membrane is believed to be a driving force in accelerating the release of non-polar DMPBD from the blend matrix. Contrarily, the release of DMPBD was delayed by the more hydrophobic nature of PLA. Although both membranes showed different release rates, their cumulative release eventually reached 100%. This high release may well arise from the large surface-area-to-volume ratio and the highly accessible interconnected pores of the fibrous membranes. Thus, the liquid medium can thoroughly penetrate the membrane. Therefore, the active compound can be released effectively from the matrix. The faster release of the active compound from the blend membrane will be beneficial for wound dressing applications to exert immediate local anesthetic and analgesic on wounds. On the other hand, slower release from the PLA membrane will facilitate transdermal application to prolong the pain-relieving and anti-inflammatory effects on the muscles [28]. This indicates that the blending of different proper polymers can modulate the properties of the material as well as tailor their applications.

To explain the release mechanism, the Ritger and Peppas model was used to fit the release data. The correlation coefficients (*R*^2^) signifying the goodness of the curve fitting were found to be 0.9923 and 0.9905 for the PLA/PEO blend and PLA fibrous membranes, respectively, revealing that the release kinetics of DMPBD were well-fitted to the Ritger and Peppas model. The calculated kinetics exponent (*n*) value was used to categorize the release profile. When *n* is lower than 0.5, the release is governed by a Fickian diffusion mechanism. When *n* is equal to 1.0, the release follows a swelling-controlled mechanism (case II transport). When *n* is between 0.5 and 1.0, the release is defined as an anomalous non-Fickian transport. It combines the swelling-controlled drug release with diffusion. The *n* value was found to be 0.46 for the PLA-based membrane, suggesting that the release of DMPBD was diffusion-controlled. The release depends on the concentration gradient of the drug between the release media and the polymer matrix [27]. In the case of the PLA/PEO blend membrane, the *n* value was 0.53, meaning that the blend exhibited an anomalous (non-Fickian) transport mechanism, in which the release was caused by diffusion and matrix swelling [36] due to the presence of hydrophilic PEO. The combination of both phenomena explains the faster release of DMPBD from the fiber blend membrane.

### 3.3. Antibacterial Test

**Figure 6:**
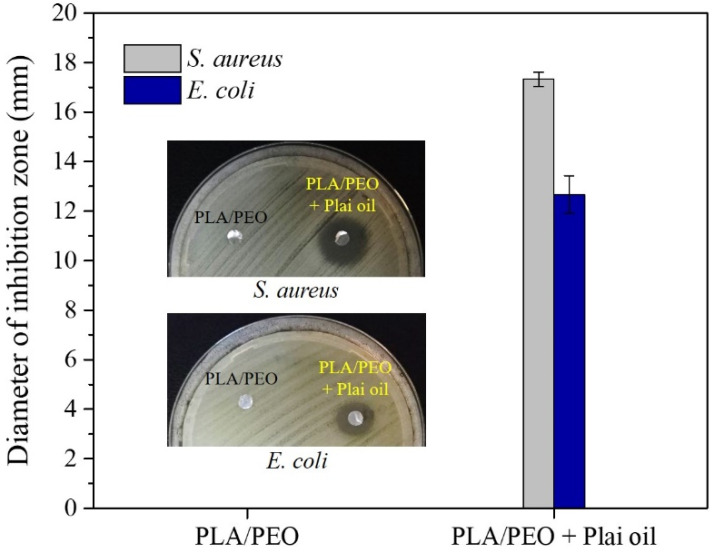
Antibacterial activity of PLA/PEO and essential oil-loaded PLA/PEO fiber blend membranes against *S. aureus* and *E. coli*.

Antibacterial activity of the fiber blend membranes was tested against two representative bacterial strains (i.e., *S. aureus*, *E. coli*) typically found in wound infection by the disk diffusion method. The fibrous membranes were mounted and incubated on the MHA agar plates for 24 h. Then, the clear inhibition zones were measured. In Figure 6, the PLA/PEO fiber blend, as a control, shows no inhibition zones against both bacterial strains, but the oil-loaded membrane exhibits the clear zones with the diameters of 17.33 ± 0.29 and 12.67 ± 0.76 mm against *S. aureus* and *E. coli*, respectively. This indicates that the *Plai* oil is responsible for the inhibition rather than the PLA/PEO blend. Therefore, the prepared *Plai* oil-loaded fiber blend membrane can be considered an alternative antibacterial wound dressing.

### 3.4. In Vitro Cytotoxicity

**Figure 7:**
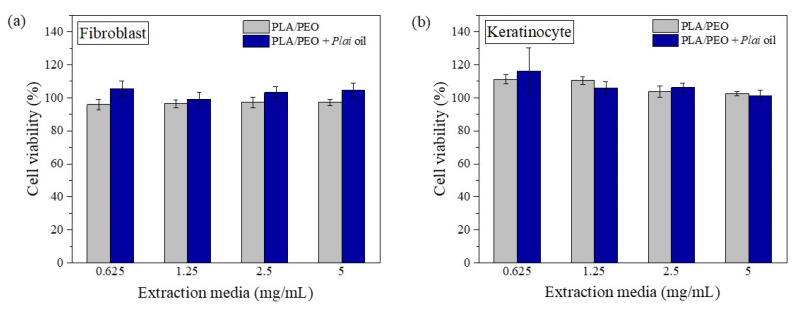
Cell viability (%) of (**a**) human dermal fibroblast and (**b**) human keratinocyte after 24 h exposure to the different concentrations of extraction media of the PLA/PEO and *Plai* oil-loaded PLA/PEO fiber blend membranes.

The indirect in vitro cytotoxicity study of the PLA/PEO blend fiber membrane with and without *Plai* oil was carried out through MTS assay on human dermal fibroblast and keratinocyte cells (HaCat). The membranes were cut into pieces and immersed in PBS buffer for 24 h to obtain the extract media at 0.63, 1.25, 2.5, and 5 mg/mL. After 24 h incubation of the tested cells to the extraction media, the cell viability was evaluated, as shown in Figure 7. A cell viability higher than 80% is considered non-toxic in this test. The viability of both fibroblast and keratinocyte cells after treatment with the extraction media of both membranes at every concentration was in the range of 95–116%, indicating that both materials are non-toxic to the tested cells. This suggests that the present *Plai* oil-loaded fiber blend membrane is safe for use as a wound dressing material.

## 4. Conclusions

In this study, *Plai* essential oil with analgesic, anti-inflammatory, and antibacterial activities was successfully loaded into the electrospun PLA/PEO fiber blend. The membrane obtained consisted of smooth fibers with diameter in the nanometer range. The hydrophilicity of the membrane can be simply modulated by blending PLA with PEO at different weight ratios. Although the membrane was hydrophilic, the *Plai* oil entrapment efficiency and physical stability against moisture in the air were still high, implying that the blend is a good platform for the loading of *Plai* oil. The hydrophilic fiber blend membrane loaded with *Plai* oil initially showed a fast release of DMPBD, followed by a sustained release in the following hours. This will be beneficial for wound care to exert immediate local anesthetic and analgesic on wounds and to maintain these effects for several hours. As the fibrous membrane had a large surface to volume ratio and highly interconnected macropores, the liquid medium could thoroughly impregnate the membrane. As such, the complete release of DMPBD was as expected. Further in vitro antibacterial and indirect cytotoxicity tests also showed that the *Plai* oil-loaded fiber blend membrane could inhibit the growth of both *S. aureus* and *E. coli* and exhibited no toxicity to human skin cells. This suggests that the present fiber blend membrane has promising potential for use in a new generation of analgesic and antibacterial wound dressings.

## Figures and Tables

**Table 1:** Mean fiber diameters of electrospun membranes.

Fiber Membranes	Mean Diameter (µm)
8% PLA/PEO (90:10)	0.53 ± 0.12^a^
8% PLA/PEO (80:20)	0.54 ± 0.09^a^
8% PLA/PEO (70:30)	0.57 ± 0.12^a^
10% PLA/PEO (90:10)	0.61 ± 0.14^b^
10% PLA/PEO (80:20)	0.62 ± 0.12^b^
10% PLA/PEO (70:30)	0.64 ± 0.14^b^
12% PLA/PEO (90:10)	0.71 ± 0.13^c^
12% PLA/PEO (80:20)	0.72 ± 0.12^c^
12% PLA/PEO (70:30)	0.74 ± 0.11^c^
12% PLA/PEO (90:10) + *Plai* oil	0.67 ± 0.12^d^
12% PLA/PEO (80:20) + *Plai* oil	0.68 ± 0.13^d^
12% PLA/PEO (70:30) + *Plai* oil	0.70 ± 0.12^d^

The statistically significant differences (*p* < 0.05) are indicated with different superscript letters.

## Data Availability

Not applicable.

## References

[1] Koontongkaew S., Poachanukoon O., Sireeratawong S., Dechatiwongse Na Ayudhya T., Khonsung P., Jaijoy K., Soawakontha R., Chanchai M. (2014). Safety evaluation of Zingiber cassumunar Roxb. rhizome extract: Acute and chronic toxicity studies in rats. Int. Sch. Res. Not..

[2] Pongprayoon U., Tuchinda P., Claeson P., Sematong T., Reutrakul V., Soontornsaratune P. (1997). Topical anti-inflammatory activity of the major lipophilic constituents of the rhizome of Zingiber cassumunar. Part II: Hexane extractives. Phytomedicine.

[3] Chongmelaxme B., Sruamsiri R., Dilokthornsakul P., Dhippayom T., Kongkaew C., Saokaew S., Chuthaputti A., Chaiyakunapruk N. (2017). Clinical effects of Zingiber cassumunar (Plai): A systematic review. Complement. Ther. Med..

[4] Cheechareoan S., Pathanawiriyasirikul T., Manmee C., Janpol K. (2016). Efficacy of Plai cream in adult patients with muscle strain: A randomized, double-blind, placebo-controlled trial. J. Med. Assoc. Thail..

[5] Leelarungrayub J., Manorsoi J., Manorsoi A. (2017). Anti-inflammatory activity of niosomes entrapped with Plai oil (Zingiber cassumunar Roxb.) by therapeutic ultrasound in a rat model. Int. J. Nanomed..

[6] Panthong A., Kanjanapothi D., Niwatananant W., Tuntiwachwuttikul P., Reutrakul V. (1997). Anti-inflammatory activity of compound D {(E)-4-(3′,4′-dimethoxyphenyl)but-3-en-2-ol} isolated from Zingiber cassumunar Roxb. Phytomedicine.

[7] Masuda T., Jitoe A., Mabry T.J. (1995). Isolation and structure determination of cassumunarins A, B, and C: New anti-inflammatory antioxidants from a tropical ginger, Zingiber cassumunar. J. Am. Oil Chem. Soc..

[8] Ozaki Y., Kawahara N., Harada M. (1991). Anti-inflammatory effect of Zingiber cassumunar Roxb. and its active principles. Chem. Pharm. Bull..

[9] Jeenapongsa R., Yoovathaworn K., Sriwatanakul K.M., Pongprayoon U., Sriwatanakul K. (2003). Anti-inflammatory activity of (E)-1-(3,4-dimethoxyphenyl) butadiene from Zingiber cassumunar Roxb. J. Ethnopharmacol..

[10] Prakatthagomol W., Sirithunyalug J., Okonogi S. (2012). Comparison of antibacterial activity against food-borne bacteria of Alpinia galanga, Curcuma longa, and Zingiber cassumunar. CMU J. Nat. Sci..

[11] Boonyanugomol W., Kraisriwattana K., Rukseree K., Boonsam K., Narachai P. (2017). In vitro synergistic antibacterial activity of the essential oil from Zingiber cassumunar Roxb against extensively drug-resistant Acinetobacter baumannii strains. J. Infect. Public Health.

[12] Taechowisan T., Suttichokthanakorn S., Phutdhawong W.S. (2018). Antibacterial and cytotoxicity activities of phenylbutanoids from Zingiber cassumunar Roxb. J. Appl. Pharm. Sci..

[13] Alven S., Buyana B., Feketshane Z., Aderibigbe B.A. (2021). Electrospun nanofibers/nanofibrous scaffolds loaded with silver nanoparticles as effective antibacterial wound dressing materials. Pharmaceutics.

[14] Stoica A.E., Chircov C., Grumezescu A.M. (2020). Nanomaterials for wound dressings: An up-to-date overview. Molecules.

[15] Keshvardoostchokami M., Majidi S.S., Huo P., Ramachandran R., Chen M., Liu B. (2021). Electrospun nanofibers of natural and synthetic polymers as artificial extracellular matrix for tissue engineering. Nanomaterials.

[16] Tanzli E., Ehrmann A. (2021). Electrospun nanofibrous membranes for tissue engineering and cell growth. Appl. Sci..

[17] Politi S., Carotenuto F., Rinaldi A., Di Nardo P., Manzari V., Albertini M.C., Araneo R., Ramakrishna S., Teodori L. (2020). Smart ECM-based electrospun biomaterials for skeletal muscle regeneration. Nanomaterials.

[18] Li G., Zhao M., Xu F., Yang B., Li X., Meng X., Teng L., Sun F., Li Y. (2020). Synthesis and Biological Application of Polylactic Acid. Molecules.

[19] Im S.H., Im D.H., Park S.J., Chung J.J., Jung Y., Kim S.H. (2021). Stereocomplex Polylactide for Drug Delivery and Biomedical Applications: A Review. Molecules.

[20] Boey J.Y., Mohamad L., Khok Y.S., Tay G.S., Baidurah S. (2021). A Review of the Applications and Biodegradation of Polyhydroxyalkanoates and Poly(lactic acid) and Its Composites. Polymers.

[21] Honarbakhsh S., Pourdeyhimi B. (2011). Scaffolds for drug delivery, part I: Electrospun porous poly(lactic acid) and poly(lactic acid)/poly(ethylene oxide) hybrid scaffolds. J. Mater. Sci..

[22] Heunis T., Bshena O., Klumperman B., Dicks L. (2011). Release of bacteriocins from nanofibers prepared with combinations of poly(d,l-lactide) (PDLLA) and poly(ethylene oxide) (PEO). Int. J. Mol. Sci..

[23] Abid S., Hussain T., Nazir A., Zahir A., Ramakrishna S., Hameed M., Khenoussi N. (2019). Enhanced antibacterial activity of PEO-chitosan nanofibers with potential application in burn infection management. Int. J. Biol. Macromol..

[24] Eskitoros-Togay Ş.M., Bulbul Y.E., Tort S., Demirtaş Korkmaz F., Acartürk F., Dilsiz N. (2019). Fabrication of doxycycline-loaded electrospun PCL/PEO membranes for a potential drug delivery system. Int. J. Pharm..

[25] Wang B., Li H., Yao Q., Zhang Y., Zhu X., Xia T., Wang J., Li G., Li X., Ni S. (2016). Local in vitro delivery of rapamycin from electrospun PEO/PDLLA nanofibers for glioblastoma treatment. Biomed. Pharmacother..

[26] Dai R., Lim L.-T. (2015). Release of allyl isothiocyanate from mustard seed meal powder entrapped in electrospun PLA–PEO nonwovens. Food Res. Int..

[27] Locilento D.A., Mercante L.A., Andre R.S., Mattoso L.H.C., Luna G.L.F., Brassolatti P., Anibal F.d.F., Correa D.S. (2019). Biocompatible and biodegradable electrospun nanofibrous membranes loaded with grape seed extract for wound dressing application. J. Nanomater..

[28] Tonglairoum P., Chuchote T., Ngawhirunpat T., Rojanarata T., Opanasopit P. (2014). Encapsulation of Plai oil/2-hydroxypropyl-β-cyclodextrin inclusion complexes in polyvinylpyrrolidone (PVP) electrospun nanofibers for topical application. Pharm. Dev. Technol..

[29] Wongkanya R., Teeranachaideekul V., Makarasen A., Chuysinuan P., Yingyuad P., Nooeaid P., Techasakul S., Chuenchom L., Dechtrirat D. (2020). Electrospun poly(lactic acid) nanofiber mats for controlled transdermal delivery of essential oil from Zingiber cassumunar Roxb. Mater. Res. Express.

[30] Ritger P.L., Peppas N.A. (1987). A simple equation for description of solute release I. Fickian and non-fickian release from non-swellable devices in the form of slabs, spheres, cylinders or discs. J. Control Release.

[31] Negut I., Grumezescu V., Grumezescu A.M. (2018). Treatment strategies for infected wounds. Molecules.

[32] Zaarour B., Zhu L., Jin X. (2020). A Review on the secondary surface morphology of electrospun nanofibers: Formation mechanisms, characterizations, and applications. Chem. Sel..

[33] Liu W., Huang C., Jin X. (2014). Tailoring the grooved texture of electrospun polystyrene nanofibers by controlling the solvent system and relative humidity. Nanoscale Res. Lett..

[34] Athanasoulia I.-G., Tarantili P.A. (2017). Preparation and characterization of polyethylene glycol/poly (L-lactic acid) blends. Pure Appl. Chem..

[35] Lim J.S., Park K., Chung G.S., Kim J.H. (2013). Effect of composition ratio on the thermal and physical properties of semicrystalline PLA/PHB-HHx composites. Mater. Sci. Eng. C.

[36] Fu Y., Kao W.J. (2010). Drug release kinetics and transport mechanisms of non-degradable and degradable polymeric delivery systems. Expert Opin. Drug Deliv..

